# The emerging role of aptamers in targeted cancer immunotherapy

**DOI:** 10.1016/j.omton.2025.201110

**Published:** 2025-12-31

**Authors:** Sai Zhu, Ruiling Xu, Haodong Xu, Yu Wang, Lu Wang, Na He, Hong-Hui Wang, Xiaolei Ren

**Affiliations:** 1 Department of Orthopedics, The Second Xiangya Hospital, Central South University, No.139 Middle Renmin Road, Changsha, Hunan 410011, P.R. China; 2 Hunan Key Laboratory of Tumor Models and Individualized Medicine, The Second Xiangya Hospital of Central South University, Changsha, Hunan 410011, P.R. China; 3 College of Biology, Hunan University, Changsha, Hunan 410082, P.R. China; 4 Hunan Research Center of the Basic Discipline for Cell Signaling, Changsha, Hunan 410082, P.R. China; 5 Hunan Engineering Research Center of AI Medical Equipment, The Second Xiangya Hospital of Central South University, Changsha, Hunan 410011, P.R. China; 6 Greater Bay Area Institute of Precision Medicine (Guangzhou), Fudan University, Guangzhou, Guangdong, 511458, P.R. China

**Keywords:** MT: Regular issue, aptamer, bispecific aptamer, targeted therapy, chimeric forms

## Abstract

Immunotherapy has revolutionized cancer treatment by harnessing the immune system against malignancies; however, traditional antibody-based therapies are hampered by high production costs, immunogenicity, and off-target effects. Aptamers—short, single-stranded DNA or RNA molecules—have emerged as a promising alternative due to their high affinity and specificity, low immunogenicity, ease of chemical synthesis, and versatile structural modifications. These properties position aptamers as powerful tools for targeted drug delivery, immune modulation, and precision cancer therapy. This review highlights recent advances in aptamer-based cancer immunotherapy, focusing on their structural evolution, responsiveness to the tumor microenvironment, and roles in regulating the immune checkpoint. We highlight the development of monospecific, bispecific, and multispecific aptamers, emphasizing their applications in T cell activation, cytokine regulation, and tumor-targeted immune modulation. Additionally, we explore the role of aptamer-based chimeric systems in immunotherapy, including aptamer-small interfering RNA (siRNA) conjugates, aptamer-nanomaterial hybrids, aptamer-drug complexes, and aptamer-exosomes conjugates. Despite progress, challenges remain, such as the need for greater *in vivo* stability, improved delivery strategies, and optimized multispecific designs. Future research efforts should focus on refining next-generation immune checkpoint-targeting aptamers and expanding their applications in combination immunotherapies. With continued innovation, aptamer-based therapeutics hold the potential to transform cancer immunotherapy, offering safer, more effective, and highly specific treatment options.

## Graphical abstract

**Figure:**
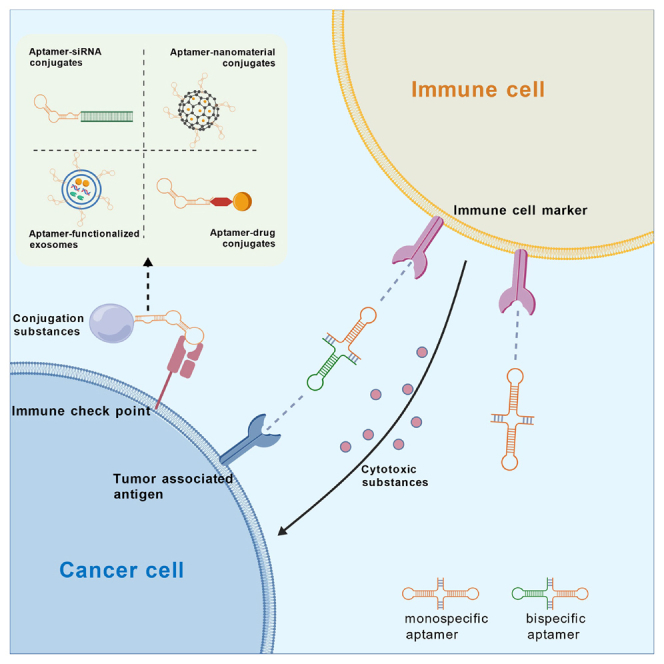

## Teaser

Aptamers, with superior specificity and modifiability, overcome antibody limitations in cancer immunotherapy by enabling tumor-responsive targeting and immune checkpoint modulation. The aptamer-based chimeric systems show therapeutic promise, yet require enhanced stability and delivery optimization for clinical translation.

## Introduction

World Health Organization has predicted that cancer would be the first or second cause of death for people before the age of 70 in most countries in the world.^1^ Cancer poses a significant obstacle to extending the human lifespan. Humans have developed a variety of cancer treatments, such as radiotherapy, chemotherapy, surgery, and immunotherapy.^2^^,^^3^^,^^4^ Among these treatments, cancer immunotherapy has brought revolutionary changes to oncology by transforming traditional therapeutic paradigms. Therefore, there is an urgent need to develop innovative therapeutic strategies that can minimize adverse effects while maintaining therapeutic efficacy. The human immune system comprises both innate and adaptive components, which play crucial roles in protecting individuals from diseases.^5^ However, tumor cells can evade recognition by the immune system through certain mechanisms, including the loss or alteration of tumor antigenicity, the regulation of cytokines, and the production of immunosuppressive molecules.^6^ Immunotherapy’s core is re-activating the patient’s immune system to recognize and destroy tumor cells.^7^ Currently, widely used immunotherapies include cancer vaccines,^8^ adoptive cell immunotherapy,^9^^,^^10^ and monoclonal antibody therapy.^11^

Cancer vaccines are artificially synthesized by combining tumor-specific antigens or tumor-associated antigens (TAAs) with adjuvants or using dendritic cells as delivery vehicles to prepare vaccine formulations, which are then introduced into the patient’s body to activate the patient’s immune response and kill the tumor,^12^^,^^13^ such as human papillomavirus vaccine^14^ and Sipuleucel-T (Provenge) vaccine.^15^ Moreover, adoptive cell immunotherapy involves collecting immune-active cells from tumor patients, amplifying, genetically engineering, and functionally identifying these cells *in vitro* and then infusing them back into the patient to directly kill tumor cells or stimulate an immune response to indirectly kill tumor cells. Several types of typical adoptive immunotherapy include Chimeric Antigen Receptor T cells (CAR-T),^9^ T Cell Receptor–engineered T cells (TCR-T),^16^ and Tumor-Infiltrating Lymphocytes (TIL) therapy.^17^ Until now, the Food and Drug Administration (FDA) has approved the launch of six CAR-T products, primarily used for treating hematological malignancies.^18^ Monoclonal antibodies are highly homogeneous antibodies produced by a single B cell clone, targeting only a specific antigenic epitope.^19^ They can regulate the immune system to treat cancer by targeting TAAs,^20^ immune checkpoints,^21^ or co-stimulatory receptors.^22^^,^^23^ Among these treatment methods, monoclonal antibody therapy is widely used in cancer treatment. The FDA and other agencies have approved 91 cancer antibodies.^11^ However, monoclonal antibodies have side effects, such as autoimmune diseases, acute allergic reactions, and infections,^24^ referred to as immune-related adverse events (irAEs). Therefore, finding new strategies with fewer side effects to enhance the effectiveness of immunotherapy is becoming increasingly important.

**Figure 1:**
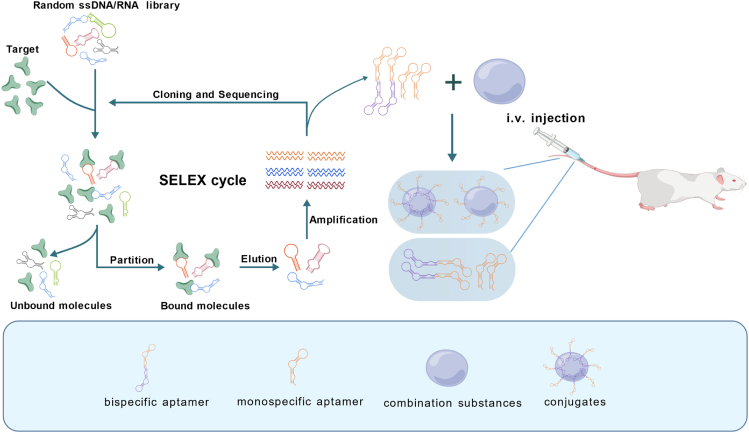
Schematic representation of the SELEX procedure and aptamer-based therapeutic modalities This schematic illustrates SELEX, a key technology for aptamer discovery. The SELEX procedure begins with a random single-stranded DNA or RNA library, which is incubated with a specific target. Bound aptamers are separated from unbound sequences, followed by elution, amplification (PCR for DNA or quantitative reverse-transcription PCR for RNA), and iterative rounds of selection (typically 8–20 cycles) to enrich for high-affinity sequences. The iterative selection process refines high-affinity oligonucleotides, which can then be engineered into therapeutic constructs, including monospecific, bispecific, and chimeric aptamers for targeted immunotherapy *in vivo*. This figure is created with BioGDP.com (https://BioGDP.com).

The aptamer is a small oligonucleotide sequence or short peptide obtained through *in vitro* screening, which can bind with the various corresponding ligands with high affinity and strong specificity. Therefore, they are usually called “chemical antibodies.”^25^^,^^26^ Aptamers are usually screened out through the systematic evolution of ligands by exponential enrichment (SELEX). The process of this technology mainly consists of four steps (i.e., binding, partition, elution, and amplification) and utilizes molecular biology techniques to interact oligonucleotide libraries with target substances (such as nucleic acids, proteins, and even cells), allowing oligonucleotide sequences that specifically bind to the target substance to be enriched through repeated amplification and screening (Figure 1).^27^^,^^28^

There are several advantages to aptamers compared to antibodies (Table 1). First, aptamers exhibit high specificity and affinity for target molecules screened *in vitro*, encompassing a broad spectrum that includes organic dyes, amino acids, proteins, antibiotics, and even cells, pathogenic bacteria, viruses, and tissues. In contrast, the targets of antibodies are limited to immunogenic molecules.^29^ The minimum dissociation constant between the aptamer and the target can be as low as the picomolar level. Their strong bonding occurs due to the pairing of specific complementary bases within the strand, as well as through electrostatic and hydrogen bonding interactions. This results in stable three-dimensional structures, which depend on the diverse structures and spatial conformations of single-stranded nucleic acids.^28^^,^^30^ On the other hand, owing to their relatively small molecular size, aptamers can recognize and bind to unique structural domains or epitopes located within spatially constrained areas on protein surfaces—targets that are often inaccessible to much larger full-length antibody molecules. Although single-chain variable fragments are relatively small and can be assembled to form bispecific or multispecific antibodies, their size remains a limitation for generating certain multispecific formats. In contrast, aptamers offer advantages in terms of engineering flexibility and lower production costs. Furthermore, aptamers are inherently low in immunogenicity. This characteristic primarily stems from their oligonucleotide nature, as these molecules are not easily presented by the major histocompatibility complex, thereby hindering the activation of T cell-dependent immune responses, which are essential for generating high-affinity neutralizing antibodies.^31^ More importantly, aptamers are produced by chemical synthesis, which does not require the use of living organisms, allowing them to be facilely synthesized in batches and at low cost.^28^ Finally, flexible chemical modification strategies can be applied to synthesizing aptamers to enhance their stability and targeting ability. For example, in the sugar ring of single-stranded nucleic acids, various chemical groups are often introduced or removed from the 2-OH position.^32^^,^^33^

**Table 1:** Comparison of characteristics: aptamer vs. antibody

Characteristic	Aptamers	Antibodies
Chemical nature	Single-stranded DNA or RNA	Proteins (immunoglobulins)
Size	6–66 kDa (20–220 nucleotides)	150–180 kDa
Production & preparation	*In vitro* chemical synthesis, controllable process	*In vivo* biological immunization, relies on animals
Production time	Short (weeks), rapid optimization	Long (months), complex optimization
Batch-to-batch variation	Virtually none, high synthesis precision	Present, due to individual animal differences
Cost	Low for large-scale synthesis	Relatively high production cost
Stability	High, tolerant to heat and denaturants, transportable at room temperature	Low, prone to denaturation, often requires cold chain transport
Target range	Broad (proteins, small molecules, ions, cells, etc.)	Relatively narrow (primarily immunogenic molecules)
Immunogenicity	Typically none or very low	High, may cause immune response
Modification & labeling	Flexible, specific groups can be precisely incorporated during synthesis	More complex, may affect structure and activity
Tissue penetration	Good, small molecular size, penetrates tissues easily	Poor, large molecular size, limited penetration
Affinity/specificity	High affinity and specificity (can distinguish a single methyl group or chirality)	High affinity and specificity
Platform maturity	Relatively novel, fewer commercial reagents and platforms	Highly mature, extensive research and diagnostic platforms

Aptamers have emerged as powerful tools in contemporary biomedical research, demonstrating remarkable versatility across disease diagnosis, targeted therapy, biosensor development, and other applications.^34^^,^^35^^,^^36^ This review provides an in-depth examination of the key advancements in aptamer-mediated immunotherapy over the past decade, with a particular focus on their diverse applications not only as standalone molecules but also as components of aptamer-based chimeras for exploring their translational potential in immune-mediated strategies.

### The role of specificity and valency in aptamer therapeutics

**Figure 2:**
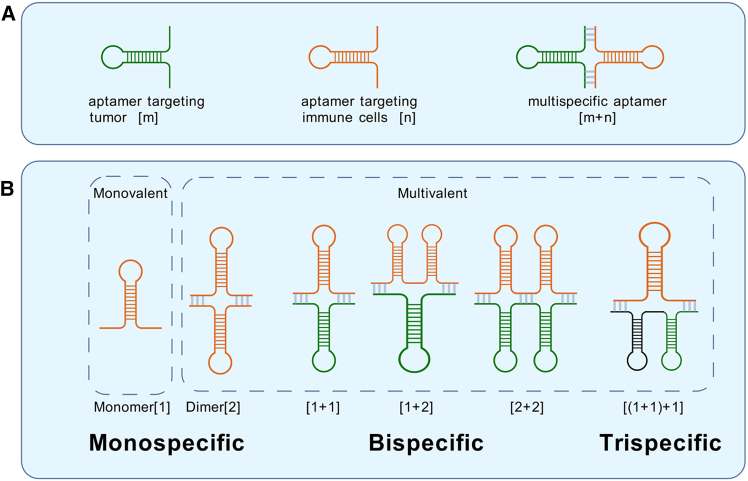
Classification of aptamer specificity and valency The figure illustrates the classification of aptamers based on specificity and valency within the context of cancer immunotherapy, providing a framework for their molecular engineering strategies. Aptamers can be designed as monospecific, bispecific, or trispecific constructs, targeting tumor cells and/or immune cells with varying receptor valency. (A) Definition of specificity: [m] represents aptamers targeting tumor cells, while [n] represents aptamers targeting immune cells. Multispecific aptamers, denoted as [m + n], can simultaneously bind multiple targets. (B) Classification of valency: monospecific aptamers can be monovalent (single binding site) or multivalent (multiple binding sites on the same target cell type). Bispecific aptamers engage both tumor and immune cell targets and are categorized based on their binding valency, such as [1 + 1], [1 + 2], and [2 + 2]. Trispecific aptamers—such as [(1 + 1) +1]—are conceptualized to bind two different tumor targets and one immune cell receptor, enhancing combinatorial targeting capabilities. This figure is created with BioGDP.com (https://BioGDP.com).

While the efficacy of an aptamer is influenced by multiple parameters, such as binding kinetics, structural stability, and pharmacokinetics,^37^ its rational design often hinges on two fundamental and programmable attributes: specificity and valency, which ensure precise and strong target engagement. For the purpose of this review, specificity refers to the type of targets, while valency refers to the number of binding sites per aptamer molecule. Clarifying and classifying aptamers based on these two attributes establishes a clear framework for understanding their diverse functional roles in cancer immunotherapy. Monospecific aptamers can only bind one type of target. A bispecific aptamer is a synthetic single-stranded DNA or RNA molecule that can simultaneously and specifically bind to two different targets, such as two different proteins, cell surface receptors, or small molecules.^38^^,^^39^ Trispecific aptamers, which could bind three distinct targets, e.g., two different cancer cell-specific targets and one type of immune cell-specific target, have not been reported yet. Valency primarily defines the number of target molecules engaged. Monovalent aptamer refers to an aptamer that targets a single receptor. Furthermore, a multivalent aptamer is composed of multiple aptameric domains designed to bind to multiple copies of a single target, which can be on the same or different cells. Therefore, aptamers can be divided into monospecific monovalent, monospecific multivalent, and bispecific multivalent ones.^40^ The relationship between the specificity and valency of the aptamer is described in detail in Figure 2. A deeper understanding of specificity and valency relationships in aptamers will facilitate the design of more effective therapeutics, particularly in multi-target immunotherapy applications.

### Monospecific aptamers targeting key immune and tumor markers

Monospecific aptamers targeting distinct immune-related receptors and cytokines represent the first step toward precise cancer immunotherapy. By precisely engaging immune-modulating receptors or tumor-associated antigens, these aptamers can enhance immune activation, block inhibitory signals, or selectively deliver therapeutic payloads. This section explores key monospecific aptamers, their molecular targets, and their therapeutic applications, laying the groundwork for understanding their potential in cancer treatment. The types and applications of reported monospecific aptamers are summarized in Table 2.

**Table 2:** Monospecific aptamers for cancer immunotherapy

Aptamer	Target	Nature	Reference	Application
**Co-stimulatory receptor**				

M12-23	4-1BB	RNA	McNamara et al.^41^	Agonist
apt9.8, 9C7T	OX-40	RNA	Dollins et al.^42^; Pratico et al.^43^	Agonist
CD28apt2,CD28apt7	CD28	RNA	Pastor et al.^44^	Agonist
CD40Apt1	CD40	RNA	Soldevilla et al.^45^	Agonist
Apt8a	ICOS	RNA	Soldevilla et al.^46^	Agonist

**Co-inhibitory receptors**				

MP7	PD-1	DNA	Prodeus et al.^47^	Antagonist
PD4S	PD-1	DNA	Gao et al.^48^	Antagonist
PL1	PD-L1	DNA	Gao et al.^49^	Antagonist
aptPD-L1	PD-L1	DNA	Lai et al.^50^	Antagonist
Del60	CTLA-4	RNA	Santulli-Marotto et al.^51^	Antagonist
aptCTLA-4	CTLA-4	DNA	Huang et al.^52^	Antagonist

**Cytokines or cytokine receptors**				

AON-D21	C5a	RNA	Ajona et al.^53^	Binding
NOX-A12	CXCL12	RNA	Vater et al.^54^; Zboralski et al.^55^	Binding
mNOX-E36	CCL2	RNA	Kulkarni et al.^56^	Binding
CIL-6A6-1, SOMAmers	IL-6/sIL-6R	DNA	Yao et al.^57^; Gupta et al.^,^^58^	Binding
aptTNF-α-PEG, VR11	TNF-α	DNA	Lai et al.^59^; Orava et al.^60^	Binding
anti-EGFR aptamer	EGFR	RNA	Wan et al.^61^; Wan et al.^62^	Binding
RNV66	VEGF	DNA	Edwards et al.^63^	Binding

ICOS, inducible synergistic co-stimulation molecules; C5a, complement 5a; CCL2, C-C motif ligand 2; sIL-6R, soluble form of IL-6 receptor; TNF-α, tumor necrosis factor-α; EGFR, epidermal growth factor receptor.

### Immune-targeting aptamers for immune modulation

#### Aptamers targeting co-stimulatory receptors in T cells

T cells play a pivotal role in the immune system, safeguarding the body against cancer cells and viruses.^64^ Due to the ability of T cells to target and clear tumor cells with minimal damage to normal cells, most current research focuses on enhancing the immune response capability of T cells.^65^ Studies have demonstrated that a second signal, known as a co-stimulatory signal, is necessary to activate T cells.^66^ Several co-stimulatory receptors have been discovered, including CD28, 4-1BB, OX-40, and CD40.

McNamara et al. developed a specific aptamer (M12-23) to target the 4-1BB molecule on the cell surface. *In vitro*, experiments have shown that this aptamer can stimulate the proliferation ability of CD8 T cells and induce the secretion of IFN-γ. They also conducted animal experiments and successfully observed a decrease in tumor volume in a DBA-2 mouse model, which was equivalent in efficacy to antibody therapy. Meanwhile, they also found that monovalent aptamers cannot generate co-stimulatory signals, whereas multivalent aptamers can, which may be related to the cross-linking required for the 4-1BB receptor to function.^41^ Interestingly, Benaduce et al. demonstrated that the combination of 4-1BB aptamer with radiotherapy significantly improved the treatment efficacy with fewer side effects of breast cancer compared to single-therapy regimens (aptamer or antibody monotherapy).^67^ This indicated that the combination of aptamers with conventional therapy had potential clinical value in patients with poor response to single therapy.

Pastor et al.^44^ confirmed that the aptamer CD28Apt7 targeting CD28 can promote cellular immunity by detecting the release of interleukin-2 (IL-2) and γ-interferon. Combining the Id vaccine with the aptamer and detecting the titer of anti-Id antibodies concluded that the aptamer could promote humoral immunity. Finally, they treated BALB/c mice inoculated with A20 lymphoma cells with a combination of Id vaccine and aptamer and found significant inhibition of tumor growth and improved survival rate. Similarly, they also verified that the monovalent form of the aptamer could not generate a co-stimulatory effect but weakened it, while the dimeric form could generate co-stimulatory signals. Collectively, based on the findings from McNamara et al.^41^ and Pastor et al.,^44^ it is suggested that only multivalent aptamers can effectively generate T cell co-stimulatory signals, whereas monovalent forms cannot.

Moreover, it has been established that CD40 and CD40L are a pair of co-stimulatory molecules that can promote the immune response. Soldevilla et al. successfully demonstrated that CD40Apt1, an aptamer targeting CD40, could induce the activation of B lymphocytes and dendritic cells. At the same time, they connected the CD40 aptamer and small hairpin RNA (shRNA), which inhibited SMG1 from forming a CD40Apt-SMG1-shRNA chimera. In BALB/c mice with lymphoma, it is evident that this chimera significantly improves the survival of the mice. Furthermore, the CD40 agonist aptamer can promote the conditions for antigen-presenting cell activation, enhance tumor antigenicity, and facilitate bone-marrow aplasia recovery.^45^ The successful development of monospecific aptamers targeting co-stimulatory receptors like 4-1BB, CD28, and CD40 demonstrates the significant potential of aptamers as potent immune agonists.

Together, these studies establish that multivalency is a critical design principle for aptamer-mediated immune agonism, essential for achieving the required receptor cross-linking.

#### Immune checkpoint inhibition via aptamers

Checkpoint molecules suppress immune function. Inhibiting checkpoint molecules can boost endogenous anti-cancer responses. Tumor cells evade T cell cytotoxicity by upregulating checkpoint ligands, such as cytotoxic T-lymphocyte-associated protein 4 (CTLA-4) and programmed cell death ligand 1 (PD-L1).^68^

Santulli Marotto et al. reported that the 35-nucleotide-long functional aptamer derivative, Del 60, which binds to CTLA-4, could inhibit the growth of mouse melanoma, especially its tetramer derivative, Del 60 tetramers. Its ability to enhance T cell proliferation is 10–20 times higher than that of its monomer form.^51^ In subsequent studies, Huang et al. reported a novel high-affinity CTLA-4-antagonizing DNA aptamer, aptCTLA-4. It possessed a fair serum half-life without modification, which could enhance T cell activity and inhibit the growth of mouse TC-1, Lewis lung cancer, and CT26 colon cancer cells.^52^ Leveraging the fact that programmed cell death protein 1 (PD-1)/PD-L1 interaction inhibits the secretion of IL-2, Prodeus’ team found that the anti-PD-1 aptamer (MP7) could remove the inhibition on IL-2 secretion. Moreover, after treating colon cancer mice with polyethylene glycol (PEG) MP7, which is more stable in blood serum, the volume and quantity of tumors were significantly reduced.^47^

Gao et al. modified the aptamer targeting PD-1 by conjugating it with cholesterol, thereby improving its stability, and naming it aptamer PD4S. They found that the anti-colon cancer effect of PD4S was higher than that of the PBS-treated group, but not as good as that of the antibody-treated group. The team believed this may be due to the rapid clearance of the subcutaneous injection.^48^ The team isolated a high-affinity and specific anti-PD-L1 aptamer, PL1. It could also inhibit the progression of mouse colon cancer by inhibiting the PD-1/PD-L1 pathway, and its effect may be related to the reduction of microvasculature within the tumor. Compared to PD4S, the inhibitory effect of PL1 on tumor growth is comparable to that of the antibody-treated group, likely due to improvements in the cell-SELEX method and the use of engineered cells expressing PD-L1 as target cells.^49^

In addition to chemically modifying the aptamer to enhance its stability, other types of nucleic acids can also be used to generate more stable aptamers. When using threose nucleic acid to screen for the aptamer N5 against PD-L1, compared to the reported DNA aptamer AptPDL1,^50^ it was more effective in inhibiting the binding of PD-1/PD-L1. Additionally, the aptamer could be effectively concentrated at the tumor site to effectively inhibit tumor growth. Collectively, the successful development of antagonistic aptamers targeting key checkpoints like CTLA-4 and PD-1/PD-L1 validates their clinical potential for future cancer therapy.^69^

### Cytokine-targeting aptamers in cancer therapy

Cytokines play an important role in the growth and progression of tumors. C-X-C motif chemokine ligand 12 (CXCL12) can bind to C-X-C chemokine receptor 4/C-X-C chemokine receptor 7 (CXCR4/CXCR7) on the cell surface, promoting tumor growth, invasion, and metastasis.^54^ NOX-A12 was a PEGylated nucleic acid aptamer drug developed by NOXXON Pharma targeting CXCL12. It was constructed using non-natural L-nucleotides. Compared to natural D-nucleotides, it had significant advantages in plasma stability and anti-nucleolytic properties due to its non-recognition by most plasma nucleases.^70^^,^^71^ Previous studies have shown that compared to radiotherapy alone, radiation therapy combined with NOX-12 could significantly prolong the lifespan of rats with glioblastoma multiforme.^72^ The clinical research of NOX-12 has entered phase 2 trials and has excellent potential to become a new drug for cancer treatment.^73^ Aptamers engaged in clinical trials for cancer treatment are summarized in Table 3.

**Table 3:** Aptamers for cancer therapy in clinical trials

Aptamer	Target	Form	Indication	Phase	NCT number
AS1411	Nucleolin	DNA	Solid tumors	Phase 1, completed	NCT00881244
			RCC	Phase 2, unknown status	NCT00740441
			AML	Phase 2, completed	NCT00512083
			Refractory or relapsed AML	Phase 2, terminated	NCT01034410
AST-201	GPC3	DNA	Advanced OC	Phase 2, not yet recruiting	NCT05794659
			Solid tumors	Phase 1, recruiting	NCT06687941
AM003	LAG3	DNA	Solid tumors	Phase 1, completed	NCT06258330
AON-D21	CD88	DNA	Lung cancer	Phase 2, recruiting	NCT05962606
NOX-A12	CXCL12	RNA	Relapsed MM	Phase 2, completed	NCT01521533
			Relapsed CLL	Phase 2, completed	NCT01486797
			CRC, PAAD	Phase 1/2, completed	NCT03168139
			GBM	Phase 1/2, active; not recruiting	NCT04121455
			MSS metastatic pancreatic cancer	Phase 2, not yet recruiting	NCT04901741
^68^Ga-Sgc8	PTK7	DNA	CRC	Phase 1, unknown status	NCT03385148
[^68^Ga] NOTA-Sgc8	PTK7	DNA	BLCA	Not applicable, recruiting	NCT06005116
[^68^Ga] NOTA-DNA multivalent Sgc8	PTK7	DNA	BLCA	Not applicable, recruiting	NCT06763354

RCC, renal cell carcinoma; AML, acute myeloid leukemia; GPC3, glypican-3; OC, ovarian cancer; LAG3, lymphocyte activation gene-3; CD88, cellular adhesion molecule 88; MM, multiple myeloma; CLL, chronic lymphocytic leukemia; CRC, colorectal cancer; PAAD, pancreatic adenocarcinoma; GBM, glioblastoma; MSS, microsatellite-stable; PTK7, protein tyrosine kinase 7; BLCA, bladder cancer; NOTA, 2,2′,2′′-(1,4,7-triazacyclononane-1,4,7-triyl)triacetic acid.

In summary, the development of monospecific aptamers has demonstrated promising therapeutic potential in modulating immune responses and targeting tumor-specific markers, establishing their viability for targeted immune-modulating therapy. However, their effectiveness is often limited by target accessibility, binding affinity, and *in vivo* stability. To enhance their clinical applicability, future research should focus on chemical modifications for improved pharmacokinetics, optimizing aptamer-target interactions, and developing innovative delivery strategies. While monospecific aptamers have shown promise, bispecific aptamers further enhance therapeutic efficacy by simultaneously engaging multiple cell types. The following section explores the structural design and functional applications of bispecific aptamers in cancer immunotherapy.

### Bispecific aptamers enhancing dual-targeted immunotherapy

Building upon the advantages of monospecific aptamers, bispecific aptamers have been developed to enhance therapeutic efficacy by simultaneously engaging tumor cells and immune cells. These engineered nucleic acid aptamers are designed to bridge immune effectors and cancer cells, promoting immune-mediated tumor clearance. In recent years, efforts have focused on optimizing bispecific aptamers to enhance immune cell recruitment, activation, and tumor-targeting efficiency (Table 4). A key strategy in cancer immunotherapy involves the design of most bispecific aptamers that function as adaptors, redirecting and activating immune effector cells at tumor sites by concurrently binding to a surface marker on cancer cells and a receptor (such as co-stimulatory receptor 4-1BB) on immune cells. Therefore, to standardize the classification of these bispecific aptamers, a naming convention based on specificity and valency has been proposed. In this system, bispecific aptamers are denoted as [m + n], where [m] corresponds to the valency of the aptamer module targeting tumors and [n] corresponds to the valency of the aptamer module targeting immune cells (Figure 2).^40^ This structured nomenclature provides a clear framework for distinguishing immunomodulating bispecific aptamer designs, aiding in the rational development of next-generation aptamer-based immunotherapies.

**Table 4:** Bispecific aptamers for dual-target immunotherapy

Tumor target (aptamer name) [nature]	Immune target (aptamer name) [nature]	Reference	Application
**1 + 1**			

c-Met (CLN0003) [DNA]	CD16α (CLN0020) [DNA]	Boltz et al.^74^	Induces ADCC
Hepatocellular carcinoma (TSL11a) [DNA]	CD16α (CLN0020) [DNA]	Zheng et al.^75^	Induces ADCC

**1 + 2**			

VEGF (ARC245) [DNA]	4-1BB (M12-23) [RNA]	Schrand et al.^76^	Co-stimulation
OPN (OPN-R3) [RNA]	4-1BB (M12-23) [RNA]	Wei et al.^77^	Co-stimulation
PSMA (A10) [RNA]	4-1BB (M12-23) [RNA]	Pastor et al.^78^	Co-stimulation
MRP1 (MRP1Apt) [RNA]	CD28 (CD28Apt7) [RNA]	Soldevilla et al.^38^	Co-stimulation

**2 + 2**			

MUC1 (MA3) [DNA]	CD16α (CLN0020a) [DNA]	Li et al.^79^	Induces ADCC

ADCC, antibody-dependent cell-mediated cytotoxicity; OPN, osteopontin; PSMA, prostate-specific membrane antigen; MRP1, multidrug resistance protein 1; MUC1, mucin-1.

[1 + 1] represents an aptamer that simultaneously targets both an immune target and a cancer target. CD16 is expressed on the surface of various immune cells, including NK cells, T cells, monocytes, and macrophages, providing a potential aptamer target.^79^ In order to overcome the problem of easy degradation of the aptamer *in vivo*, Zheng et al. designed a Y-shaped DNA aptamer, assembled form the TLS11a aptamer, which exhibits a specific affinity for hepatocellular carcinoma and a single specific aptamer targeting CD16, featuring a Y-shaped skeleton. Confocal microscopy imaging revealed that this Y-shaped Ap promoted NK cell aggregation at the site of hepatocellular carcinoma, thereby enhancing the antitumor effect of NK cells and significantly reducing the tumor growth rate in mice.^75^

[1 + 2] is the most reported type of bispecific aptamers.^38^^,^^76^^,^^77^ To address autoimmune pathologies linked to immune modulation, Schrand et al. developed a bispecific aptamer targeting vascular endothelial growth factor (VEGF) and 4-1BB (CD137). This aptamer binds to the broadly expressed VEGF in stromal tissues, allowing immune co-stimulation to focus on tumor lesions. By conjugating this aptamer with two agonistic 4-1BB oligonucleotide aptamers, the complex targets the co-stimulatory receptor on activated CD8+ T cells. It could significantly enhance the antitumor effect of the GVAX vaccine, which was better than the single specific aptamer mixture injected with VEGF and 4-1BB. This finding indicates that bispecific aptamers can indeed enhance immune effects by connecting two target cells rather than simply combining the two aptamers.^76^

[2 + 2] has been developed to enhance the therapeutic effect of cancer immunotherapy. Mucin1 (MUC1) is associated with the occurrence and development of tumors, especially adenocarcinoma, and is a potential target for cancer diagnosis and treatment.^80^ In a recent study, two aptamers targeting MUC1 and two targeting CD16 were linked through three 60-nt DNA spacer regions rich in A and C bases. Under a fluorescence microscope, this bivalent bispecific aptamer (BBiApt) promoted the recruitment of CD16-positive immune cells around MUC1+ cells. Cell viability testing suggested that this aptamer enhances the killing effect of immune cells on tumor cells. Unfortunately, the research team did not conduct *in vivo* experiments to verify the targeting effect of the aptamer, possibly due to the unresolved issue of the aptamer’s instability *in vivo*.^79^

Currently, there are no reports of trispecific or multi-specific aptamers. Increasing the valence and specificity of aptamers can enable them to bind to different types of cells with higher affinity. However, it is unclear which valence and specific design has the best targeting effect, as there is no direct comparison of the targeting effects of aptamers such as [1 + 1] and [1 + 2]. Due to the varying expression levels of target receptors in different cells, spatial hindrance, and other factors, an upper limit to affinity gain may increase valence or specificity, but not enhance the targeting effect.^40^^,^^81^ Therefore, further research is necessary to investigate the optimal design configurations for multispecific aptamers, thereby elucidating their mechanisms of action and potential applications.

### Aptamer-based chimeric therapeutics integrating versatile functionalities

Beyond standalone aptamer therapies, recent advancements have led to the development of chimeric aptamer-based systems. By integrating aptamers with small interfering RNA (siRNA), nanoparticles, or chemotherapeutic agents, these hybrid systems enhance drug stability, target specificity, and therapeutic efficacy (Table 5). Crucially, these platforms enhance cancer immunotherapy by enabling the highly specific, localized delivery of immunomodulatory payloads. When serving as a targeting moiety, the aptamer acts as an intelligent guiding module, directing conjugated siRNA or drug molecules to accurately recognize and accumulate at tumor sites and efficiently mediating their cellular uptake. This process directly addresses the critical challenge of intracellular delivery for therapeutics that must act within the cell, thereby reducing systemic toxicity and circumventing systemic drug resistance.^93^ Consequently, aptamer-based targeted delivery systems present a highly promising platform for achieving efficient and precise disease treatment. Generally speaking, aptamers can be coupled with drugs/siRNA/exosomes/nanomaterials through covalent and non-covalent means. The types of aptamers-drug conjugation strategy can be classified into two modes: physical conjugation or intercalation, achieved through covalent or noncovalent bonding, and chemical linking (aptamers can be covalently linked to target molecules using various chemical reactions, including click chemistry, amide bond formation, and thiol-maleimide coupling).^94^ There are two main methods for attaching siRNA to aptamers: (1) non-covalently conjugate siRNA with aptamers through a connector such as streptavidin and packaging RNA and (2) covalently link siRNA to aptamers, forming an aptamer-siRNA chimera.^93^^,^^95^ Exosome-aptamer coupling is achieved via exogenetic modification, including membrane anchoring, streptavidin-biotin bridges, and direct chemical conjugation (e.g., click chemistry), or via endogenetic engineering such as glycosylphosphatidylinositol (GPI) anchoring.^96^^,^^97^ The method of coupling nanoparticles with aptamers involves both covalent coupling (i.e., amide bonds) and non-covalent coupling (i.e., electrostatic interactions, hydrophobic interactions, hydrogen bonding, and avidin biotin bridging). In addition, nanoparticles can also encapsulate drugs and siRNA to achieve therapeutic effects against tumors.^93^ The coupling methods of aptamer-based chimeric have already been described in detail elsewhere.^94^^,^^95^^,^^97^^,^^98^

**Table 5:** Chimeric aptamer therapeutics and applications

Chimera	(Aptamer) target	Coupling substances	Reference	Application
**siRNA**				

4-1BB-Smad4 conjugate	(4-1BB aptamer) 4-1BB	siRNA	Puplampu-Dove et al.^82^	Immune activation
EpCAM-AsiCs	(EpCAM aptamer) EpCAM	siRNA (Upf2, Parp1, Cd47, and Mcl1)	Zhang et al.^83^	Induce tumor neoantigens

**Nanocarriers**				

X-polymers	(CD28Apt7) CD28, (Del60) CTLA-4	Three-dimensional junction scaffold	Bai et al.^84^	Aptamer carrier
sgc8c/hp-Au NP	(sgc8c) PTK7	Gold nanoparticle(Au NP)	Luo et al.^85^	Drug carrier
TCL-SPION-Apt	(A10) PSMA	TCL-SPION	Wang et al.^86^	Drug carrier
PSMA-aptamosomes	(xPSM-A9) PSMA	Liposome	Baek et al.^87^	Promote targeted drug delivery

**Drug**				

NucA-PTX	(AS1411) nucleolin	Paclitaxel	Li et al.^88^	Promote targeted drug delivery
PEG-APT-DOX	(MUC1-apt) MUC1	Doxorubicin	Tan et al.^89^	Promote targeted drug delivery

**Exosomes**				

DOX-Apt-Exo	(AS1411) nucleolin	Exosomes	Hosseini et al.^90^	Promote targeted drug delivery
EXO_apt_-siRNA	(E3) prostate cancer cells	Exosomes	Han et al.^91^	Promote targeted siRNA delivery
sgc8-Exos-D	(sgc8) PTK7	Exosomes	Zou et al.^92^	Promote targeted drug delivery

4-1BB, TNF receptor superfamily member 9 (TNFRSF9) or CD137; EpCAM, epithelial cell adhesion molecule; Upf2, up-frameshift 2; Mcl1, myeloid cell leukemia-1; Parp1, poly(ADP-ribose) polymerase 1; PSMA, prostate-specific membrane antigen; MUC1, mucin 1; PTK7, protein tyrosine kinase 7.

**Figure 3:**
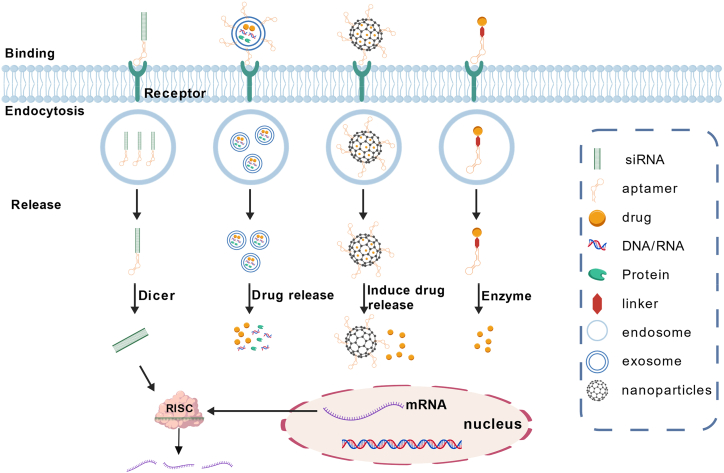
Mechanisms of chimeric aptamer therapeutics for targeted cancer therapy The schematic illustrates the mechanisms by which aptamer-based chimeric systems facilitate targeted cancer therapy. Aptamers specifically bind to tumor cell surface receptors, enabling cellular uptake via endocytosis, followed by intracellular release of therapeutic agents. The four major chimeric strategies are depicted. (1) Aptamer-siRNA chimeras: once internalized, the siRNA separates from the aptamer under the action of DICER, leading to the formation of the RISC, which subsequently mediates gene silencing. (2) Extracellular vesicle-based delivery: engineered exosomes and other extracellular vesicles transport drugs, nucleic acids, or proteins, releasing them into the cytoplasm upon uptake. (3) Nanoparticle conjugates: aptamer-functionalized nanoparticles deliver encapsulated drugs intracellularly, where they are released under specific stimuli (e.g., pH and enzymatic cleavage). (4) ApDCs: the aptamer-drug linker is enzymatically cleaved, leading to drug dissociation and activation of its therapeutic function. This figure is created with BioGDP.com (https://BioGDP.com).

These chimeric constructs leverage the unique binding specificity of aptamers while incorporating siRNA, nanocarriers, small-molecule drugs, or exosomes, expanding their functionality for targeted gene silencing, controlled drug delivery, and immune modulation. The structural diversity and cellular mechanisms of these aptamer-based chimeras are systematically illustrated in Figure 3, highlighting their role in next-generation precision cancer therapies.

### Aptamer-siRNA conjugates for targeted gene silencing

siRNA holds promise for cancer gene therapy by degrading specific mRNA through the RNA-induced silencing complex (RISC) complex to suppress gene expression.^99^ However, the clinical translation of siRNA is hampered by various factors, including drug delivery pathways, biological barriers, exosmosis, tissue or cell uptake, and endosomal escape.^95^ Aptamer-siRNA conjugates have emerged as an effective approach to overcome these obstacles and facilitate precise siRNA delivery. Besides, aptamer-siRNA conjugates can merge the precision targeting of aptamers with the gene-silencing capability of siRNA, creating a powerful platform for cancer immunotherapy. These constructs enable tumor-specific knockdown of immunosuppressive targets to enhance anti-tumor immunity.

In one study, an AS1411 aptamer targeting nucleolin was conjugated to SMG1-siRNA to inhibit nonsense-mediated mRNA decay. This AS1411-SMG1 chimera triggered tumor inflammation and significantly improved responses to immune checkpoint blockade across multiple tumor models.^100^ Another innovative design combined AS1411 with PD-L1 siRNA in a multifunctional nanoparticle (Chimera/PEI/Gln/β-CD/DOX) for lung squamous cell carcinoma treatment. This system effectively silenced PD-L1 in tumor cells, activating T cells and CD8+ T cells by blocking PD-1/PD-L1 immunosuppression. *In vivo* results showed markedly reduced tumor growth alongside significantly enhanced T cell and CD8+ T cell infiltration, demonstrating potent immune activation.^101^ At the same time, an aptamer can be coupled with multiple siRNAs to simultaneously deliver multiple siRNAs, blocking the transmission of multiple signaling pathways and better inhibiting tumor progression.^83^

### Aptamer-nanomaterial conjugates for enhanced drug delivery

Nanomaterials exhibit good biocompatibility and an enhanced permeability and retention effect, which can prolong the circulation time of drugs in the bloodstream and increase drug absorption.^102^ However, nanomaterials lack specificity for therapeutic targets and may lead to systemic cell damage, which has been an urgent issue to address.^103^

The conjugation of aptamers with nanomaterials represents a cutting-edge approach in cancer immunotherapy, integrating the high specificity of aptamers with the multifunctional loading and delivery capabilities of nanomaterials to achieve precise immune modulation at the tumor site and synergistically enhance anti-tumor immune responses. Ren et al. developed an acid-responsive nanoassembly (DNA-PAE@BAY-876) co-loading glycolysis inhibitor BAY-876 with PD-L1/CTLA-4 antagonistic aptamers for triple-negative breast cancer. This system simultaneously inhibits PD-L1 glycosylation and reprograms regulatory T cells (Tregs), effectively reversing immunosuppression and enhancing checkpoint blockade therapy.^104^ In another study, Zhang et al. created PD-L1 aptamer-functionalized metal-organic framework nanoparticles (M@O-A) encapsulating oxaliplatin. This platform combines photodynamic therapy, chemotherapy, and immunotherapy, demonstrating potent anti-tumor effects against both primary and distant tumors, while showing minimal systemic toxicity in irAE-mimic models.^105^ Lai et al. constructed PD-L1 aptamer-decorated albumin nanoparticles loaded with fexofenadine (PDL1-NP-FEXO), where the aptamer enables the nanoparticle target PD-L1-expressing tumor cells, while FEXO reduces immunosuppressive M2 macrophages. This combination significantly enhanced tumor suppression without increasing toxicity in mouse models.^106^

### Aptamer-drug conjugates for immunotherapy

Aptamer-drug conjugates (ApDCs) are playing an increasingly important role in tumor immunotherapy. Their mechanism of action extends far beyond simple targeted drug delivery, achieving synergistic effects between chemotherapy and immunotherapy. The core of this strategy lies in the fact that aptamers not only serve as guidance systems for precisely delivering chemotherapeutic agents but also act as immune modulators that activate anti-tumor immune responses by blocking immunosuppressive signals or remodeling the tumor microenvironment. For instance, the PD-L1 aptamer-gemcitabine conjugate (PD-L1-GEMs) developed by Hu et al. exemplifies this strategy, where the aptamer not only enables tumor-specific drug delivery but also acts as an immune checkpoint inhibitor. This combination leads to synergistic efficacy against bladder cancer by coupling direct cytotoxicity with restored T cell activity.^107^ Similarly, the research by Cong et al. focuses on another critical immunosuppressive target—galectin-1. They conjugated the AP74 aptamer with the chemotherapeutic drug βIZP to construct AP74-βIZP. The conjugation not only delivered the cytotoxic drug but also enhanced the anti-tumor immune response by promoting the infiltration of CD4+/CD8+ T cells and elevating pro-inflammatory cytokines, creating a more favorable tumor microenvironment for cancer cell elimination.^108^ Furthermore, Zuo et al. demonstrated that AS1411-camptothecin conjugate nanoparticles (AS-CPT-4 NPs) exert a complex immune-reprogramming function. Beyond its targeting role, the AS1411 aptamer inhibits the IKK/NF-κB pathway and reshapes the tumor immune microenvironment by favoring CD8+ over CD4+ T cell infiltration, thereby overcoming chemoresistance and immunosuppression.^109^

### Aptamer-functionalized exosomes for targeted drug delivery

Exosomes are a type of extracellular vesicles, which are elliptical nanovesicles secreted by living cells. They have advantages of small size, high safety, and good stability. Multiple studies have confirmed that extracellular vesicles can be used as a delivery tool to deliver drugs for treating tumors, improving the effectiveness of tumor treatment.^110^^,^^111^

The coupling system of aptamers and exosomes provides an innovative platform for enhancing the targeting and efficacy of cancer immunotherapy by integrating the natural delivery properties of exosomes with the precise recognition capability of aptamers. Oncolytic virus is a type of tumor-killing virus with efficient replication ability; oncolytic virus therapy is one of the most promising immunotherapy directions.^112^ In the study of Bahreini et al., AS1411 aptamer-decorated exosomes were co-loaded with an oncolytic virus and doxorubicin (ExomiR-CVB3/DoxApt). This system shielded the therapeutic cargo, enabled targeted delivery to breast cancer cells, and demonstrated potent anti-tumor efficacy by synergistically combining virotherapy, chemotherapy, and immune activation in the tumor microenvironment.^113^ Separately, a CD133-targeting aptamer-functionalized exosome (Ex-apt@LuCXB) was designed to deliver a novel photosensitizer for immuno-photodynamic therapy. This nanoplatform effectively targeted cancer stem cells and, upon irradiation, eradicated tumors in mouse models of liver and breast cancer by synergizing antiangiogenesis with a robust photoinduced immune response.^114^

Together, chimeric aptamer-based strategies represent a promising frontier in cancer immunotherapy. Future research should focus on optimizing conjugation techniques, *in vivo* stability, and clinical translation to fully realize the potential of these approaches in precision medicine.

## Challenges and possible solutions

Despite their great potential, the path from aptamers as laboratory tools to clinical therapeutics is fraught with several key challenges: stability, *in vivo* delivery efficiency, production costs, and the effectiveness gap between *in vitro* screening and *in vivo* application. Although these issues can be partially addressed through strategies such as chemical modification, PEGylation, and binding with nanocarriers (such as exosomes), these challenges remain the main obstacles to its successful clinical translation.

### During selection and development

#### Nuclease degradation and stability

Unmodified natural DNA/RNA aptamers are highly susceptible to rapid degradation by nucleases present in biological fluids. This results in an extremely short half-life, leading to their swift inactivation.^26^

#### Chemical modification and optimization

Chemical modifications (e.g., incorporation of 2′-fluoro or 2′-O-methyl ribose) aimed at enhancing stability and binding affinity constitute a complex, costly, and time-consuming process. Furthermore, inappropriate modifications can disrupt the critical three-dimensional structure necessary for target binding.^28^

#### SELEX technology bottlenecks

##### Selection complexity

When employing traditional SELEX against complex targets (such as whole cell membranes and intact viruses), it can be challenging to efficiently enrich aptamers that bind specifically to disease-relevant epitopes.^28^^,^^36^^,^^115^

##### Non-specific binding

It is notoriously difficult to eliminate sequences that bind non-specifically to other components within the selection system (e.g., the solid-phase matrix itself) rather than the intended target.^26^

##### Differences in internal performance

The significant disparity between the *in vitro* selection conditions and the complex *in vivo* physiological environment often results in aptamers demonstrating high affinity in test tubes failing to maintain efficacy *in vivo*.^116^

### During preclinical and clinical translation

Rapid renal clearance: due to their relatively low molecular weight, most aptamers are rapidly filtered and cleared by the glomeruli, leading to an unacceptably short *in vivo* half-life and necessitating frequent administration.^28^

Tissue penetration and targeted delivery: the efficient delivery of aptamers to solid tumor cores remains a significant challenge.^95^

Immunogenicity: while PEGylation is used to improve pharmacokinetics of aptamer-based therapeutics, it can also induce the formation of anti-PEG antibodies in patients, which accelerates aptamer clearance and reduces efficacy.^117^^,^^118^

Synthesis and purification: while the chemical synthesis of short-chain, unmodified aptamers is generally robust and low cost, the production of long-chain, high-purity, chemically modified oligonucleotides required for clinical-grade standards (e.g., Good Manufacturing Practice) involves complex separation and purification steps, leading to exceedingly high manufacturing costs, often higher than those of traditional small-molecule drugs.^119^

### Potential solutions

To address these challenges, researchers are actively exploring multiple strategic solutions. (1) Enhancing stability and pharmacokinetic properties: chemical modifications, such as the incorporation of 2′-fluoro or 2′-O-methyl ribose, or the use of locked nucleic acids, can significantly increase nuclease resistance and extend the aptamer half-life from several hours up to 72 h. Furthermore, conjugation with PEG (PEGylation) or incorporation into nanocarriers effectively increases the molecule’s hydrodynamic radius, thereby slowing renal clearance. (2) Optimizing chemical modification: to mitigate the adverse effects associated with chemical modifications, it is imperative to optimize the structure of modifying groups while concurrently developing novel chemical modifications with enhanced biocompatibility and improved metabolic clearance. This includes the exploration of alternative “stealth” polymers beyond PEG, such as polysialic acid and polysaccharides.^28^^,^^118^^,^^120^ (3) Optimizing selection and characterization techniques: The development of novel SELEX technologies, including cell-internalization SELEX and microfluidic SELEX, enables the selection of aptamers better suited for complex physiological environments.^121^^,^^122^ Concurrently, post-SELEX optimization, leveraging bioinformatics tools and advanced characterization methods (e.g., surface plasmon resonance, bio-layer interferometry) allows for precise sequence truncation and the identification of critical nucleotides.^123^^,^^124^^,^^125^ This facilitates the generation of shorter, more stable, and higher-affinity aptamer variants.^26^ (4) Innovative delivery and combination strategies: utilizing aptamers as targeting moieties, conjugated to nanoparticles, drugs, or gene-editing tools,^126^ enables the construction of sophisticated systems such as ApDCs or smart delivery platforms.^94^ These strategies enhance targeted delivery efficiency and therapeutic specificity. The integration of stimuli-responsive elements (e.g., pH-sensitive components) further enables controlled drug release.^127^

### Conclusions and future perspectives

Targeted therapy has developed rapidly in recent years, and monoclonal antibodies targeting surface antigens have become a crucial treatment strategy for many malignant tumors.^128^ However, the production cost of antibodies is high, and there is a risk of self-immune toxicity. Aptamers have become important research objects in targeted therapy due to their wide target range, high affinity, ease of synthesis, low immunogenicity, and ease of chemical modification. The role of aptamers in immunotherapy is mainly to act as immunotherapeutic agents to regulate immune function and to couple with other substances (chemotherapy drugs, nanomaterials, siRNA, and exosomes) to exert targeted effects.

Although aptamers have potential application value in multiple fields, some unresolved issues still need to be addressed. (1) Multiple clinical trials of aptamers are underway (Table 3). However, since the FDA approved the first nucleic acid aptamer pegaptanib for market launch in 2004,^129^ there have been nearly 20 years without any other nucleic acid aptamers approved for market launch. Until 2023, the second aptamer drug, i.e., avacincaptad pegol, was approved for marketing.^130^ This indicates that research on nucleic acid aptamers remains insufficient, and further preclinical studies are needed to advance the development of aptamer-based drugs. (2) There are no relevant reports on multispecific aptamers, and it is necessary to design multispecific aptamers to compare their efficacy with known aptamers. (3) Currently, a limited number of aptamers with immune regulatory functions are available. Therefore, it is crucial to develop aptamers that target novel immune checkpoints, such as TIGIT, VISTA, and LAG-3.^25^^,^^131^

Despite existing challenges, continued research efforts focusing on novel aptamer targets, optimized chemical modifications, and robust clinical validation will accelerate the translation of aptamer-based therapies into clinical oncology.

## Acknowledgments

This work was supported by the the National Key R&D Program of China (2025YFA0923100) and the 10.13039/501100001809National Natural Science Foundation of China (22177030). This work was supported by the Program (Establishment and application of osteosarcoma organoids) of Greater Bay Area Institute of Precision Medicine (Guangzhou).

## Author contributions

S.Z., writing – original draft preparation; R.X, investigation; Y.W., resources; H.X., project administration; N.H., validation; L.W., visualization; X.R., conceptualization and funding acquisition; H.W., supervision and writing – review & editing. All authors have approved the final review and the submission.

## Declaration of interests

We declare that we have no conflict of interest.
